# Supplementary material for: FeNO 350 mL/s: Unlocking the Small Airways to Achieve Clinical Remission in Severe Asthma—A Pilot Study
Source: Adv Respir Med. 2025 Sep 17;93(5):37. doi: 10.3390/arm93050037 (PMC12452774; doi:10.3390/arm93050037)
Supplement: Supplementary file 1 [file arm-93-00037-s001.zip › arm-3800485-supplementary.pdf]

Supplementary Table S1. Excluded patients: biologic therapy, age, sex, and reason for exclusion.

Pt#	Biologic therapy	Age (years)	Sex	Reason for exclusion
1	Omalizumab	26	M	FeNO350 not performed (technical failure / lack of coordination)
2	Omalizumab	65	F	FeNO350 not performed
3	Mepolizumab	69	F	FeNO350 not performed
4	Mepolizumab	35	F	FeNO350 not performed
5	Mepolizumab	61	M	FeNO350 not performed
6	Benralizumab	54	F	FeNO350 not performed
7	Benralizumab	76	M	FeNO350 not performed
8	Benralizumab	33	F	FeNO350 not performed
9	Benralizumab	55	M	FeNO350 not performed
10	Benralizumab	26	F	FeNO350 not performed
11	Benralizumab	65	M	FeNO350 not performed
12	Benralizumab	33	M	FeNO350 not performed
13	Benralizumab	57	M	FeNO350 not performed
14	Benralizumab	64	M	FeNO350 not performed
15	Dupilumab	56	F	FeNO350 not performed
16	Dupilumab	59	F	FeNO350 not performed
17	Dupilumab	53	F	FeNO350 not performed
18	Tezepelumab	30	F	FeNO350 not performed
19	Tezepelumab	67	M	Missing follow-up
20	Tezepelumab	60	M	FeNO350 not performed
21	Tezepelumab	49	M	Missing follow-up
22	Tezepelumab	27	M	FeNO350 not performed

23	Tezepelumab	64	M	Missing follow-up
24	Tezepelumab	19	F	Missing follow-up
25	Tezepelumab	60	F	FeNO350 not performed
26	Tezepelumab	60	F	Missing follow-up
27	Tezepelumab	50	M	FeNO350 not performed
28	Tezepelumab	64	F	FeNO350 not performed
29	Tezepelumab	64	M	FeNO350 not performed
30	Tezepelumab	72	M	FeNO350 not performed
31	Benralizumab	64	M	FeNO350 not performed

Supplementary Table S1. Excluded patients during the enrollment period according to biologic therapy, age, sex, and reason for exclusion. None of the excluded patients had FeNO350 measured at both baseline and follow-up, mainly due to technical malfunction, lack of coordination, or missing 12-month follow-up.

Abbreviations:
Pt#: progressive patient number; FeNO350: fractional exhaled nitric oxide at 350 mL/s; M: male; F: female.

Supplementary Table S2. Comparison of Clinical and Functional Parameters in Severe Asthma Patients Treated with Benralizumab, Stratified by Clinical Remission Status at Baseline (T0) and After One Year (T1).

Population N=12	T0 Baseline Uncontrolled Asthma Pt (n=6)	T0 Baseline Clinical Remission Pt (n=6)	p	T1 Uncontrolled Asthma Pt (n=6)	T1 Clinical Remission Pt (n=6)	p
EXACERBATIONS (y)	0 (0;1)	0 (0;0)	0.138	0 (0;0)	0 (0;0)	1.000
HOSPITALIZATIONS (y)	0 (0;0)	0 (0;0)	1.000	0 (0;0)	0 (0;0)	1.000
INFECTIOUS EXACERBATIONS (y)	0 (0;1)	0 (0;0)	0.138	0 (0;0)	0 (0;0)	0.200
UNSCHEDULED VISITS (y)	0 (0;0)	0 (0;0)	1.000	0 (0;0)	0 (0;0)	1.000

OCS (y)	0 (0;1)	0 (0;0)	0.317	0 (0;0)	0 (0;0)	0.109
ACT	22.5 (19;25)	25 (23;25)	0.145	19.8 (15;22)	24.3 (23;25)	0.295
ACQ6	2.36 (1.8;3.8)	0.81 (0.7;0.9)	0.009*	2.78 (2.1;3.2)	0.85 (0.7;1.0)	0.005*
AQLQ	5.1 (3.3;5.7)	5.1 (3.7;5.5)	0.872	4.6 (3.2;5.8)	4.7 (3.7;5.6)	0.681
TAI	54 (54;54)	54 (53;54)	0.317	54 (54;54)	54 (53;54)	1.000
FEV1 %pred	71.5 (53;88)	71 (43;103)	1.000	72 (53;98)	91 (64;98)	0.642
FEV1 (L)	2.07 (1.7;2.5)	2.33 (1.6;3.5)	0.631	2.10 (1.8;2.6)	3.1 (1.9;3.6)	0.112
FVC %pred	93 (75;110)	88.5 (64;109)	0.810	92.3 (80;104)	101 (82;103)	0.681
FVC (L)	2.99 (2.8;3.7)	3.73 (2.8;4.3)	0.337	2.9 (2.5;3.7)	3.8 (3.4;4.7)	0.080
FEV1/FVC %	82.5 (70;93)	68.5 (54;97)	0.470	80 (70;94)	90 (68;97)	0.848
REV %	2 (2;2.3)	2 (1;2.3)	0.338	2.1 (2;2.3)	1.8 (1;2.6)	0.860
MEF75 %	55.5 (34;74)	34.5 (16;90)	0.522	56 (25;99)	49 (16;109)	0.742
MEF50 %	39.5 (16;59)	21.5 (13;92)	0.522	41.6 (16;69)	43 (13;97)	0.742
MEF25 %	32.5 (16;40)	16.5 (14;67)	0.748	30 (14;48)	33 (14;70)	0.742
FeNO50 (ppb)	44.5 (6;57)	26.5 (18;30)	0.337	36 (6;60)	26 (18;36)	0.956
FeNO350 (ppb)	16.5 (10;23)	9 (7;11)	0.012*	16 (10;31)	9 (6;9.5)	0.459
WBC (cells/μL)	6260 (5540;7760)	6995 (5100;8627)	0.631	6746 (5540;9410)	7205 (5100;9880)	0.956
EOS (cells/μL)	0 (0;125)	0 (0;0)	0.140	80 (0;470)	0 (0;0)	0.084
%EOS	0 (0;2.2)	0 (0;0)	0.140	1.4 (0;8.4)	0 (0;0.6)	0.170
R5–20 ($kPa\cdot L^{-1}\cdot s^{-1}$)	0.095 (0.08;0.11)	0.12 (0.1;0.15)	0.199	0.39 (0.1;1.1)	0.1 (0.06;0.16)	0.322
Fres (Hz)	16 (13;18)	18 (15;20)	0.251	18 (15;20)	17 (12;20)	0.956
X5 ($kPa\cdot L^{-1}\cdot s^{-1}$)	-0.135 (-0.2;-0.11)	-0.115 (-0.2;-0.01)	0.168	-0.14 (-0.18;-0.11)	-0.43 (-0.9;-0.04)	0.087
AX (kPa/L)	1.3 (1;1.3)	0.5 (0.45;0.9)	0.065	1.08 (0.5;1.4)	0.76 (0.4;1.1)	0.334

Data are expressed as median (interquartile range, IQ 25–75). Comparisons were restricted to patients treated with benralizumab and stratified according to clinical remission status after one year. Mann–Whitney U test was used for continuous variables and Fisher's exact test for categorical variables. A p

value < 0.05 was considered statistically significant. Values marked with * indicate statistical significance.

Abbreviations:
N: Number of participants; Pt: Patients; y: Years; OCS: Oral Corticosteroids; ACT: Asthma Control Test; ACQ6: Asthma Control Questionnaire 6-item; AQLQ: Asthma Quality of Life Questionnaire; TAI: Test of Adherence to Inhalers; FEV1 %pred: Percentage of Forced Expiratory Volume in 1 second; FEV1 (L): Forced Expiratory Volume in liters; FVC %pred: Percentage of Forced Vital Capacity; FVC (L): Forced Vital Capacity in liters; FEV1/FVC %: Percentage of FEV1 to FVC ratio; REV %: Bronchodilator reversibility; MEF75/50/25 %: Maximal Expiratory Flow at 75%, 50% and 25% of FVC; R5–20: Difference in resistance between 5 Hz and 20 Hz; Fres: Resonant frequency; AX: Reactance area; X5: Reactance at 5 Hz; FeNO50: Fractional Exhaled Nitric Oxide at 50 mL/s; FeNO350: Fractional Exhaled Nitric Oxide at 350 mL/s; WBC: White Blood Cell count; EOS: Eosinophil count; %EOS: Percentage of eosinophils.
